# A quantitative and qualitative comparison of illumina MiSeq and 454 amplicon sequencing for genotyping the highly polymorphic major histocompatibility complex (MHC) in a non-model species

**DOI:** 10.1186/s13104-017-2654-1

**Published:** 2017-07-28

**Authors:** Haslina Razali, Emily O’Connor, Anna Drews, Terry Burke, Helena Westerdahl

**Affiliations:** 1 Department of Animal and Plant Sciences, University of Sheffield, Sheffield, S10 2TN UK; 2 Molecular Ecology and Evolution Lab, Department of Biology, Lund University, Sölvegatan 37, 223 62 Lund, Sweden

**Keywords:** MHC genes, 454, MiSeq, Amplicon sequencing

## Abstract

**Background:**

High-throughput sequencing enables high-resolution genotyping of extremely duplicated genes. 454 amplicon sequencing (454) has become the standard technique for genotyping the major histocompatibility complex (MHC) genes in non-model organisms. However, illumina MiSeq amplicon sequencing (MiSeq), which offers a much higher read depth, is now superseding 454. The aim of this study was to quantitatively and qualitatively evaluate the performance of MiSeq in relation to 454 for genotyping MHC class I alleles using a house sparrow (*Passer domesticus*) dataset with pedigree information. House sparrows provide a good study system for this comparison as their MHC class I genes have been studied previously and, consequently, we had prior expectations concerning the number of alleles per individual.

**Results:**

We found that 454 and MiSeq performed equally well in genotyping amplicons with low diversity, i.e. amplicons from individuals that had fewer than 6 alleles. Although there was a higher rate of failure in the 454 dataset in resolving amplicons with higher diversity (6–9 alleles), the same genotypes were identified by both 454 and MiSeq in 98% of cases.

**Conclusions:**

We conclude that low diversity amplicons are equally well genotyped using either 454 or MiSeq, but the higher coverage afforded by MiSeq can lead to this approach outperforming 454 in amplicons with higher diversity.

**Electronic supplementary material:**

The online version of this article (doi:10.1186/s13104-017-2654-1) contains supplementary material, which is available to authorized users.

## Background

The major histocompatibility complex (MHC) plays a key role in adaptive immunity by presenting antigens to the immune system for elimination [1]. Across all known vertebrates, the genetic region encoding the MHC is the most polymorphic described to date [2]. This polymorphism is thought to be primarily maintained by the selective advantage conferred by rare and/or multiple MHC alleles in the recognition and elimination of pathogens [3–5]. Within the fields of ecology and evolutionary biology, MHC genes have attracted a great deal of research attention, mostly due to their association with fitness-related traits, e.g. survival, lifetime reproductive success, disease resistance and fecundity [6–10]. However, the polymorphic and polygenic nature of MHC genes makes accurate genotyping a challenge [11, 12]. High-throughput sequencing (HTS) technologies offer an excellent opportunity for deep sequencing at a relatively low cost, which makes sequencing all MHC alleles simultaneously in an individual affordable and practical [13, 14]. Consequently, HTS has become the standard approach for sequencing MHC genes in non-model organisms [e.g. 15–19].

HTS comes at the cost of ‘noisy’ data: the high read numbers obtained through HTS are associated with a substantial number of artefactual reads [20–23]. An enduring challenge when working with HTS data is accurately separating true allelic reads from artefacts. This can be particularly difficult when working with a multi-gene family, such as the MHC, as the MHC region holds many similar copies of genes that cannot be amplified separately. When there are many similar alleles present in an amplicon, it can be difficult to distinguish between true allelic variants and artefactual reads based upon nucleotide differences and relative read depths, which is a central tenet of most genotyping techniques [13, 17, 24, 25].

Roche 454 amplicon sequencing (454) has been a popular choice in the study of MHC [e.g. 13, 15, 24]. However, 454 is being replaced by illumina MiSeq amplicon sequencing (MiSeq), which has now been used to accurately genotype MHC genes across many species with differing degrees of MHC complexity [17, 25, 26]. The MiSeq platform offers greater sequence coverage at a lower per-base cost than 454 and generates substantially fewer sequencing errors [27]. This lower error-rate could be particularly beneficial in study systems where individuals possess a high number of MHC alleles, as is the case in many bird species within the order Passeriformes [e.g. 25, 28–31].

To date, the focal exons in HTS studies of MHC in avian non-model organisms have been MHC class I exon 3 and MHC class IIB exon 2, as these exons encode regions responsible for antigen binding [19, 25, 28, 31–36]. MHC genotyping in Passeriformes presents a challenge, but also an excellent opportunity to test the level of diversity (number of alleles per individual) at which MiSeq outperforms 454. Although it has been suggested that MiSeq improves our ability to discern true alleles from artefacts in species with many MHC genes [25], this has yet to be tested directly. This is an important omission as there is still a great deal of research being published that has used 454 for MHC genotyping [31, 35–39]. Thus, it is valuable to determine, in species with many MHC gene copies, how 454 and MiSeq genotyping compares.

The aim of this study was to evaluate and compare, quantitatively and qualitatively, the performance of 454 and MiSeq for genotyping MHC class I alleles in house sparrows (*Passer domesticus*), as an example of a non-model species with multiple MHC gene copies. We have chosen house sparrows since they have duplicated MHC class I genes and several studies have already investigated variation in MHC class I exon 3 in this species over the last 10 years using different molecular genetic techniques [30, 32, 40, 41]. The earlier studies provides a prior expectation of the number of MHC class I alleles per individual house sparrow, as well as the expected allelic variation, e.g. allele length differences. Our dataset comprises both MiSeq and 454 data from the same 11 house sparrow families (81 individuals), plus 15% replicated samples. We have prior knowledge of primer performance, as well as the advantage of being able to use heritability within families to aid our assessment of the performance of the different techniques of MHC genotyping [30, 42]. It is important to bridge the gap between former and present molecular genetic methods, such as 454 and MiSeq, in order to be able to evaluate and interpret data from different ‘methodological eras’.

## Methods

### Samples and molecular methods

Blood samples were taken from 81 house sparrow individuals comprising 11 families: 11 adult males, 10 adult females and 60 nestlings (with nestlings often being combined from successive broods belonging to the same breeding pair). There was a minimum of three offspring in each family. The sparrow samples were obtained from a population inhabiting Lundy Island, located in the Bristol Channel (51°10°N, 4°40°W, UK) [43].

Genomic deoxyribonucleic acid (DNA) was extracted using a salt extraction [44]. The DNA concentration was then standardized to 20–25 ng/µl. Twelve individuals were chosen at random to act as replicates. Each DNA sample was split in two: half for preparation for 454 and the other half for preparation for MiSeq. The forward primer HNalla 5′-TCCCCACAGGTCTCCACAC-3′ and the reverse primer Rv3 5′-TGCGCTCCAGCTCCYTCTGCC-3′ were used to amplify a 236 to 242 bp long fragment of MHC class I exon 3 that contains the most variable portion of the peptide binding region [30, 33]. Although the use of a single primer pair may limit the number of alleles that are detected [42], for the current study the most important consideration was simply to have comparable data. In order to subsequently identify and separate the amplicons from each individual, the forward and reverse primers were each tagged with a unique 6 bp sequence combination [45] and either 454 or MiSeq adaptor sequences. Separate polymerase chain reactions (PCRs) were performed on the samples for 454 and MiSeq sequencing. PCRs were performed in 15-µl volumes containing QIAGEN Multiplex MasterMix, 10–20 ng DNA and 0.2 µM of each primer (modified for either 454 or MiSeq). PCRs were performed using the following settings: 95 °C at 15 min, then 30 cycles of 95 °C for 30 s, 65 °C for 60 s and 72 °C for 60 s, followed by a final extension at 72 °C for 10 min. PCR products were verified on a 1.5% agarose gel stained with Syber Safe (Invitrogen). Amplified DNA from sets of eight individuals was pooled (separately for 454 and MiSeq i.e. 12 pools per technique), in semi-equimolar volumes, and purified using a MinElute PCR purification kit (QIAGEN) according to the manufacturer’s instructions. The 12 pools from each of the two sets of samples were then taken in equimolar volumes to form two separate final pools (i.e. one pool for 454 and one pool for MiSeq), that were then sent either for 250 bp paired-end illumina MiSeq sequencing (illumina, Inc., San Diego, CA, USA) at the Sheffield Diagnostic Genetics Service or 454 pyro-sequencing (Roche, Branford, CT, USA) at the DNA sequencing facility at the Department of Biology, Lund University. See Galan et al. [24] and Kozich et al. [45] for detailed descriptions of the 454 and MiSeq amplicon sequencing techniques, respectively. The 454 sequencing run was conducted in 2012, whereas the MiSeq sequencing run was conducted in 2013. In the case of the 454 run, the 93 samples were in a single quadrant along with 192 samples from another study. For the MiSeq run, the 93 samples were in a single lane with 115 other samples. Prior experience of 454 sequencing of MHC-I in house sparrows [30] gave us confidence that the reads per sample, i.e. coverage, would be sufficient given 285 samples in the quadrant. For the MiSeq run, the number of reads per lane was anticipated to be so high that insufficient coverage given the number of samples was considered unlikely.

### Sequence preprocessing

In the case of the MiSeq data, the sequences were assembled, based on ≥100 bp overlaps, using FLASH [46]. Next, PRINSEQ was used to remove any sequences with a Phred quality score below Q30 [47]. Finally, sequences were demultiplexed, trimmed of their tags and primer sequences, then summarised in a table listing the read number (read depth) of each sequence in each amplicon using jMHC [48]. In the case of the 454 data, the raw fasta file was processed by jMHC in the same manner.

### Genotyping of MiSeq and 454-data

Only variants between 239 and 242 bp in length (classical MHC class I genes) were retained in the dataset in order to eliminate variants of 236 bp in length (non-classical MHC class I genes) that were co-amplified [30, 42]. In the present study we will only focus on classical MHC class I alleles, which are characterized by high nucleotide diversity and positively selected sites in exons 2 and 3 [30, 49, 50].

The degree of change (DOC) method described by Lighten et al. [17] was used for genotyping both MiSeq and 454 data as this is a widely used method in MHC studies [e.g. 18, 35, 36, 38, 51, 52]. This method uses the relative read depths of variants to distinguish true alleles from artefactual variants and enables an estimation of the number of alleles present in each individual (*A*_*i*_). An important preliminary step in this method is to perform the initial error correction step as described by Lighten et al. [17]. The error correction step increases the read depth of true alleles by assigning reads arising from artefacts to the true alleles from which they arose, i.e. the parent sequence. The parent sequence is a sequence with a high number of reads, considered a true allele. This ‘cleaning’ step increases the read depth of true variants relative to artefactual variants, aiding the later calculation of the DOC value.

Error correction was performed separately and sequentially on each amplicon. To identify variants arising from true alleles, the 50 variants with the highest read depths were aligned, separately for each amplicon, and neighbour-joining trees produced in CodonCode aligner 5.0.2 (CodonCode Corporation). These trees were used to visually assess the variants and identify possible artefacts as variants that differed from one another by just one or two nucleotides or containing homopolymers. The top 50 variants were used as this should encompass the (up to) eight classical MHC alleles previously reported in house sparrow individuals [30, 40], though even higher numbers of classical MHC alleles cannot be excluded, as well as many common PCR or sequencing errors. This method relies upon an assumption that true alleles will have higher read numbers than variants arising from artefacts.

In the case of variants differing by one or two nucleotides, the read depths of the two variants were compared. If one variant occurred at less than 50% of the read depth of the other variant then it was considered a possible artefact. In the case of the 454 data, we checked whether the same possible artefact occurred in any other amplicons. If it only occurred in one amplicon it was considered an artefact and thus deleted and the read numbers added to the parent variant. If it occurred in more than one amplicon in the 454 dataset the variant was considered a true allele. A different rule was applied to variants differing by one or two nucleotides in the MiSeq data, as illumina sequencing is more prone to generating repeatable nucleotide substitution errors, i.e. miscalling the same nucleotide repeatedly depending on the flanking nucleotide sequence [17, 53, 54]. Thus, in the MiSeq data a variant that differed from another by just one or two nucleotides was only considered to be a true allele if it always occurred at over 50% of the read depth of the other variant or was present in other amplicons without the putative parent variant. In the case of variants containing homopolymers, which are particularly common in 454 data, the variant was considered an artefact if it occurred at a lower read depth than the parent variant and did not occur in any other amplicon without the parent variant. As rare MHC alleles are expected within populations, variants occurring in a single sample were not discarded on this basis alone.

After error correction, the degree of change (DOC) value was calculated as detailed in Lighten et al. [17]. One of the key assumptions underpinning Lighten et al.’s [17] DOC method is that real alleles will be amplified at significantly higher sequencing depths than artefacts. Thus, there should be a clear difference in the rate of change (ROC) in the cumulative sequencing depth between the true allele with the lowest sequencing depth and the artefact with the highest sequencing depth. Calculations described by Lighten et al. [17] enable the DOC around each variant to be calculated as a percentage of the total change among all variants, in their study using the top 10 variants per individual on the assumption that up to eight true variants exist. In the current study the top 12 variants were used to calculate the DOC, as up to eight true alleles were expected [30, 40], but more were possible. Cumulative sequencing depth graphs were plotted in Microsoft Excel for each amplicon to enable the identification of genotypes with and without clear inflection points (Additional file 1: Figure S1). Three people independently evaluated each amplicon either as a ‘good amplicon’ with a clear inflection point or as a ‘poor amplicon’ without a clear inflection point; the latter were excluded from further analysis. The consensus requirement was that all three people should agree on whether an amplicon was ‘good’ or ‘poor’.

The mean read depths of true alleles (*A*_*i*_, as calculated from the DOC method) and the mean read depths of artefacts were calculated per amplicon for different *A*_*i*_ values. The DOC method may exclude true alleles with poor amplification efficiency [16]. However, as such alleles would be excluded from both the 454 and MiSeq data, this does not influence comparability, which is the focus of the current study.

### Quantitative comparison of MiSeq and 454

Quantitative comparisons of the performance of MiSeq and 454 were conducted by assessing the relative success rate (i.e. number of ‘good amplicons’) between the two methods. Additionally, the effect of different *A*_*i*_ values on the success rate was assessed both within and between the two techniques.

### Qualitative comparison of MiSeq and 454

Qualitative comparisons of the performance of MiSeq and 454 were conducted by assessing the match between genotypes obtained within and between these two techniques. The proportion of genotypes that matched within the 12 replicated sample pairs was calculated, separately for the MiSeq and 454 data, by dividing the number of replicate pairs that had matching genotypes by the total number of replicate pairs. The proportion of matching genotypes between the MiSeq and 454 data was assessed for the 81 individuals (i.e. replicates not included). This was calculated by dividing the number of matching genotypes by the total number of possible genotype matches. The genotypes of the chicks were also compared to those of their parents, separately for the MiSeq and 454 data, to further verify the reliability of the genotypes.

### Statistical analysis

Mann–Whitney U-tests were used to test whether there was a significant difference in the read depth between ‘good’ and ‘poor’ amplicons for each technique [55]. A Fisher’s exact test was used to investigate whether the frequency of ‘good’ amplicons was similar between the two techniques [55].

### Naming alleles

A BLAST query was performed on each allele remaining in the final dataset to check whether it had been previously identified. An allele was considered identical to a previously verified sequence only if it had 100% identity to the published sequence and was the same length. Identical alleles were given the same name as the published sequence. When alleles had 100% identity to a published sequence, but did not have 100% query coverage (i.e. they were shorter or longer than a published sequence but matched 100% in their overlapping segment), they were given the name of the published sequence followed by an ‘a’. The alleles that had not previously been reported were given species-specific names following the recommended guidelines for naming new MHC alleles [56] and uploaded to GenBank.

## Results

### Sequencing depths before and after error correction

Considerably more reads were obtained from the MiSeq run than the 454 run: 727,913 reads in total for MiSeq and 17,687 in total for 454 (total number of reads for sequences with complete tags and primers). After removal of all singleton reads the mean (±se) number of reads per amplicon was 4923 ± 99 for MiSeq and 126 ± 3 for 454. Once non-classical alleles were excluded the mean (±se) number of reads per amplicon was 2747 ± 58 for MiSeq and 66 ± 1.6 for 454. Ninety-three and 92 amplicons were successfully sequenced in the MiSeq and 454 runs, respectively. For a summary of the mean read per amplicon at each stage of data processing, see Additional file 1: Table S1.

After error correction, 24 and 17 ‘poor’ amplicons were discarded from the 454 and MiSeq datasets, respectively (454: *N* = 68 remaining amplicons, MiSeq: *N* = 76 remaining amplicons). After error correction and the removal of ‘poor’ amplicons, the mean (±se) number of reads per amplicon was 2818 ± 62 reads for MiSeq and 63 ± 2 for 454, i.e. MiSeq amplicons had a significantly higher read depth. The mean (±se) number of reads per allele was 11 ± 0.3 (range: 3–36) for 454 and 264 ± 7 (range: 65–768) for MiSeq. The total reads across all true alleles was 3711 for the 454 data and 96,924 for the MiSeq data. Thus 79% of the total reads were discarded from the 454 data (13,976 out of 17,687) whereas 87% were discarded from the MiSeq data (630,989 out of 727,913). This high percentage of discarded reads in both datasets is largely attributable to the removal of non-classical alleles (Additional file 1: Table S1). The ‘poor’ amplicons that were discarded from the dataset had lower read depths than the ‘good’ amplicons for both the MiSeq and the 454 data (Mann–Whitney U-test, *Z*_MiSeq_ = 2.82, *P* < 0.005, *Z*_454_ = 2.87, *P* < 0.005; Table 1).

**Table 1:** The number of reads (after error correction) for amplicons that were classified as ‘good’ or ‘poor’ in the MiSeq (‘good’, *N* = 76 and ‘poor’, *N* = 17) and 454 data (‘good’, *N* = 68 and ‘poor’, *N* = 24)

	Mean (±se) reads per amplicon	
	MiSeq	454
‘Good’ amplicons	2818 (62)	63 (2)
‘Poor’ amplicons	2427 (135)	53 (2)

The same 21 MHC-I alleles were found in both the 454 and MiSeq datasets (Additional file 1: Figure S2). 19 of these alleles have been previously found in other house sparrow individuals, supporting the validity of the genotyping protocol. We identified two new alleles (Pado-UA*396 and Pado-UA*397, Genbank Accession Numbers KY314123 and KY314124). There were between three and nine putative alleles per individual whereas previous studies found up to eight alleles per individual [30, 40]. The higher number of alleles per individual in the current study either reflects copy number variation between individuals, which is a common feature of MHC genes [e.g. 16, 17, 26, 57], or the relative heterozygosity/homozygosity at different loci, which cannot be disentangled from these data. Furthermore, the same alleles may be shared among loci [see also 26], making it difficult to determine how many loci are present from the number of alleles per individual [57].

### Separating alleles from artefacts in amplicons with different numbers of putative alleles

**Fig. 1:**
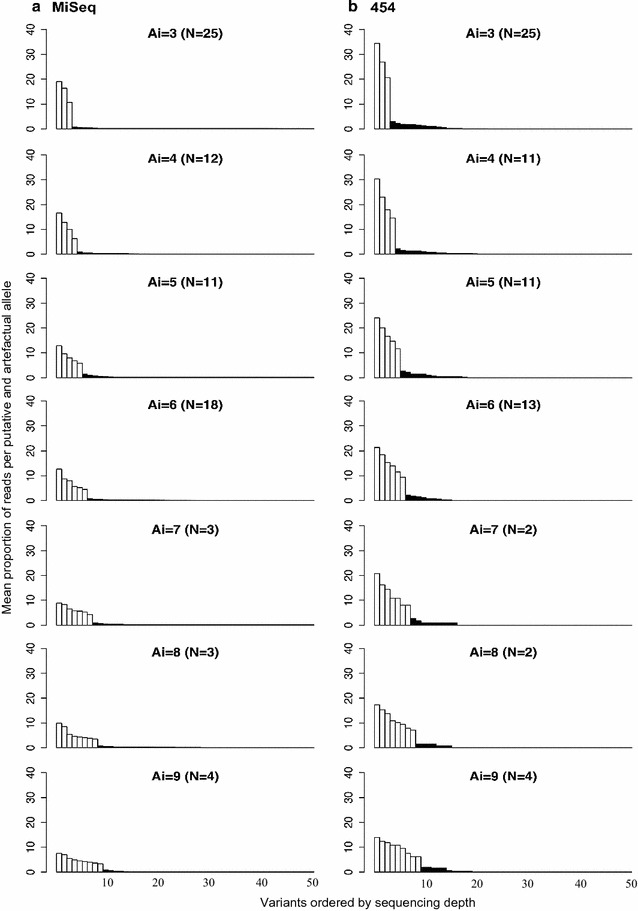
Mean proportion of reads of the first 50 variants (true and artefact alleles) in amplicons with different numbers of putative true alleles (*A*_*i*_) for the MiSeq (**a**) and the 454 (**b**) data. The mean sequencing depth for each allelic level (i.e. putative alleles ordered by depth) was calculated as the total number of reads from all successfully genotyped amplicons per allelic level divided by the total reads per amplicon. These calculations were performed separately on amplicons grouped by the number of putative alleles they possessed (*A*_*i*_ = 3 to 9 alleles). Total numbers of amplicons: *N*_MiSeq_ = 76, *N*_454_ = 68. *Grey bars* show the sequencing depths of true alleles, whereas *black bars* show the sequencing depths of artefacts

Overall, there was a clear difference in the cumulative sequencing depth between true alleles and artefacts after error correction for both the MiSeq and 454 data (Fig. 1). In the case of the MiSeq data, true alleles occurred at relative sequencing depths between 3.3 and 19.1%, whereas artefacts were observed to have much lower depths, between 0.001 and 1.4% (Fig. 1a). In the 454 data, the true alleles were observed at sequencing depths between 6.6 and 34.4%, whereas artefacts were observed at lower sequencing depths, between 0.05 and 3.0% (Fig. 1b). These percentage values for MiSeq and 454 were calculated based on the 50 variants with the highest sequencing depths and then presented as the proportion of the total reads in each amplicon in Fig. 1. In the case of the 454 data, the 50 variants represent a considerably higher proportion of the full dataset than in the MiSeq data because the raw 454 amplicons had an average of 106 variants whereas the raw MiSeq amplicons had an average of 4961 variants. Thus, the proportion of reads within the top 50 variants was higher for the 454 data than the MiSeq data, although the number of reads was much higher for MiSeq as stated above. The difference between the cumulative sequencing depth of putative alleles and artefacts decreased as the number of alleles per amplicon increased in both the MiSeq and 454 data.

### Quantitative comparison of MiSeq and 454

Among the 81 and 80 amplicons in the MiSeq and 454 data, respectively (replicates not included), there were more ‘good’ amplicons in the MiSeq data (69/81 i.e. 85%) than the 454 data (58/80 i.e. 73%). However, this difference was not statistically significant (Fisher’s exact test, *P* > 0.05).

**Fig. 2:**
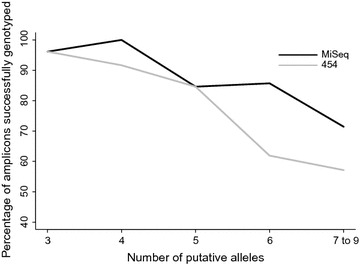
The percentage of amplicons successfully genotyped using MiSeq (*black*) and 454 (*grey*), with different numbers of true alleles (*A*_*i*_ = 3 to 9 alleles), out of the total number of individuals with each *A*_*i*_. The number of shared amplicons between MiSeq and 454 per *A*_*i*_ was: 3 = 24, 4 = 11, 5 = 8, 6 = 9, 7–9 = 2. Refer to Fig. 1 for the total number of genotyped amplicons

For both the MiSeq and 454 data, the proportion of successfully genotyped amplicons was generally lower when the number of putative alleles was higher (Fig. 2). In amplicons with six or more alleles, a lower proportion of amplicons was successfully genotyped using 454 compared to MiSeq.

### Qualitative comparison of MiSeq and 454

Among the 12 replicated sample pairs, there were seven and two ‘good’ amplicon pairs for the MiSeq and 454 data, respectively. There was 100% genotype match between the replicated amplicon pairs for both the MiSeq and 454 data.

Fifty-five individuals were successfully genotyped using both MiSeq and 454 (matching amplicons), with a mean genotype match of 98% across techniques. The 2% discrepancy was the result of a mismatch between MiSeq and 454 genotyping in a single individual (MiSeq *A*_*i*_ = 6, 454 *A*_*i*_ = 3). The number of true alleles in this individual was likely to be six given that replicated samples in the MiSeq data both had six alleles whereas in the 454 data just one replicate was successfully genotyped.

Among the 11 families that were genotyped, seven and two families, in the MiSeq and 454 data, respectively, had enough ‘good’ amplicons to enable successful genotyping of both parents and one or more chicks. All the alleles detected in the chicks were found in at least one of the parents for both the MiSeq and 454 data (MiSeq, 41 chicks; 454, 9 chicks).

## Discussion

In this study we demonstrate similar performance between MiSeq and 454 amplicon sequencing for genotyping multi-locus MHC class I genes in a non-model species (house sparrows) with up to nine different alleles per individual. We first discuss the relative use and performance of MiSeq and 454 for genotyping MHC in general. Next, we discuss the results of our quantitative and qualitative comparison of MiSeq and 454 to discover if, and at what complexity, MiSeq outcompetes 454. Finally, we briefly discuss methodological considerations for improving MHC genotyping using PCR and HTS techniques.

**Fig. 3:**
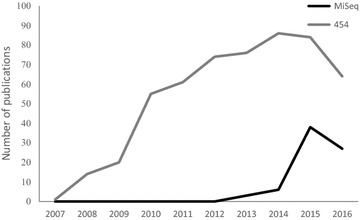
The number of publications per year mentioning 454 or MiSeq from 2007 to 2016. Numbers obtained from Google Scholar using the search terms “major histocompatibility complex” and either “MiSeq sequencing” or “454 sequencing” on 18 May 2017

Since the introduction of 250 bp paired-end sequencing made MiSeq a viable option for MHC genotyping (around 2012), there has been a gradual increase in the number of studies using MiSeq for this purpose (Fig. 3). Roche began to phase out 454 in 2015, and although it still appears to be the dominant technology in recent publications, MiSeq will replace 454 over time. 454 has been repeatedly shown to offer a reliable alternative to more traditional, non-HTS, approaches for MHC genotyping in non-model species [15, 16, 58, 59]. However, we are not aware of many direct comparisons of MiSeq and 454 for MHC genotyping. A recent study checked the congruence of MHC genotypes obtained using 454 and MiSeq in eight white-footed mice (*Peromyscus leucopus*), and found perfect agreement [60]. However, the individuals in that study had just two MHC alleles each. Ours is the first study, to our knowledge, to compare directly the performance of these two HTS methods in a species with high MHC diversity.

In the current study, quantitative comparisons came from assessing the proportion of successful amplicons within and between each method, while qualitative comparisons came from assessing genotype matching. In terms of the quantitative comparisons, a slightly lower proportion of the 454 amplicons were considered ‘good amplicons’ and successfully genotyped (73%) than the MiSeq amplicons (85%). This difference between techniques was more pronounced in amplicons with six or more alleles. Overall, the amplicons that were classified as ‘poor’ had significantly fewer reads in both the MiSeq and 454 data. This suggests that insufficient coverage led to these amplicons failing to be genotyped and, as previously stated, this was more common for the 454 data. However, as eight of the amplicons that were classified as ‘poor’ in both the 454 and MiSeq data came from the same individuals, it is likely that, in these cases, poor DNA quality prior to sequencing explains the failed genotyping.

In terms of the qualitative estimates, 454 and MiSeq were highly comparable. There was only a single case in which there was not a full genotypic match between the two methods (in an individual with *A*_*i*_ = 6). Given the large discrepancy in the number of reads between these two methods (MiSeq: 2818 ± 62, 454: 63 ± 2, mean ± se), this is somewhat surprising and indicates that, although 454 provided fewer reads, they were of high quality. Indeed, the overall quality of reads in the current study may have been slightly higher for the 454 run than the MiSeq run, given that a smaller percentage of the total reads were discarded from the 454 data compared to the MiSeq data. Additionally, it should be noted that the reads per amplicon for the 454 data in this study were fairly low. Had we achieved greater read depth per amplicon in our 454 sequencing, it is very likely that the two methods (MiSeq and 454) would have been entirely equivalent in both their qualitative and quantitative performance.

The genotypes of chicks matched expectations, given the genotypes of their parents. In this study we used the family data to validate our genotyping protocol, but pedigree information can also be a powerful tool to improve genotyping methods by enabling the identification of true alleles in poor quality amplicons [26].

Some of the methodological problems faced in HTS are PCR-based errors that occur before sequencing. When an error originates early in the PCR process an artefactual variant can achieve a high read number in the final dataset, making it more difficult to discern from the true alleles. PCR-based problems can be minimized by optimizing DNA extraction protocols [61] and by reducing the number of PCR cycles to 20–25 [62, 63], as artefact formation occurs at a higher rate with more cycles [62]. Another artefact that can occur prior to the HTS is cross-contamination when setting up the PCR [64]. In our study, some amplicons included sequences that were similar to putative alleles in other samples but were classified as artefacts due to low sequencing depth. These variants were most likely due to cross-contamination between DNA samples when setting up the PCR, a common occurrence in large multiplexing studies [17, 21]. As all 21 MHC-I alleles in this study were detected in replicated amplicons from independent PCRs, we are confident that none of these alleles are the result of contamination.

We used the DOC protocol for genotyping MHC in this study. We found that this genotyping method accurately separated putative alleles from artefacts in both the MiSeq and 454 data. The error correction stage of the DOC increases the read depth of true alleles by assigning some of the artefactual variants to their parent sequences, which facilitates accurate genotyping. Thus, it is possible that a more extensive error correction step could have enabled successful DOC genotyping in some of the ‘poor’ amplicons. Tools have recently become available [e.g. 65] that enable automated clustering of artefactual variants and true alleles, making more extensive error correction prior to performing DOC feasible. Another important methodological consideration for MHC genotyping is that of having sufficient coverage. As previously mentioned, genotyping failed in slightly more 454 samples than MiSeq samples, presumably due to insufficient coverage. Although MiSeq offers increasingly high reads per sequencing run, it may still be important to consider the number of MHC alleles expected per sample and adjust the number of samples per run accordingly [15].

## Conclusions

In conclusion, despite substantially lower reads in the 454 data, there was high agreement between the HTS methods, MiSeq and 454, on genotyping classical MHC class I genes in house sparrows. Although more of the 454 amplicons failed the genotyping procedure, of the 55 amplicons that were genotyped successfully with both MiSeq and 454, the agreement was 98%, i.e. only a single sample failed. Our findings suggest that both MiSeq and 454 are reliable techniques for assessing MHC genotypes when there is sufficient coverage, given the expected level of MHC diversity, and that the results obtained with the two methods are comparable (Additional files 2, 3).

## Additional files

**Additional file 1: Figure S1.** Depicts the relationship between cumulative read depth per variant for ‘good’ and ‘poor’ amplicons. **Figure S2.** Is an alignment of the amino acid sequences for the 21 putative alleles described by this study. **Table S1.** Is a summary of the mean reads per amplicon at different stages of the data processing.

**Additional file 2.** Additional file contains details of the number of MHC class I alleles detected in each sample in the study.

**Additional file 2.** Additional file contains details of the number of MHC class I alleles detected in each sample in the study.

## Abbreviations

MHC: major histocompatibility complex
HTS: high-throughput sequencing
MiSeq: illumina MiSeq amplicon sequencing
454: 454 amplicon sequencing
PCR: polymerase chain reaction
DNA: deoxyribonucleic acid
DOC: degree of change
ROC: rate of change
*A*_*i*_: the number of true alleles per individual
