# Supplementary material for: A quantitative and qualitative comparison of illumina MiSeq and 454 amplicon sequencing for genotyping the highly polymorphic major histocompatibility complex (MHC) in a non-model species
Source: BMC Res Notes. 2017 Jul 28;10:346. doi: 10.1186/s13104-017-2654-1 (PMC5534077; doi:10.1186/s13104-017-2654-1)
Supplement: Supplementary file 1 — Additional file 1: Figure S1. Depicts the relationship between cumulative read depth per variant for ‘good’ and ‘poor’ amplicons. Figure S2. Is an alignment of the amino acid sequences for the 21 putative alleles described by this study. Table S1. Is a summary of the mean reads per amplicon at different stages of the data processing. [file 13104_2017_2654_MOESM1_ESM.docx]

**Additional material**

**Figure S1.** An example of a ‘good’ and ‘poor’ amplicon for MiSeq and 454 data, respectively. ‘Good’ (filled) amplicons have a clear difference in the sequencing depth of true alleles and artefact variants. In ‘poor’ amplicons (open) this difference is less distinct. (a) MiSeq amplicons: examples of a ‘good’ amplicon with three real putative alleles and a ‘poor’ amplicon. (b) 454 amplicons: examples of a ‘good’ amplicon with four real putative alleles and a ‘poor’ amplicon.

**Figure S2.** Alignment of house sparrow MHC -class I amino acid sequences deduced from MiSeq and 454 amplicon sequencing. The inferred alleles had identical sequences with each method and all sequences were detected by both methods.

**Table S1.** Summary of mean (± se) reads per amplicon at each stage of data processing for the MiSeq and 454 data.

|  | Mean (± se) reads per amplicon | |
| --- | --- | --- |
|  | **MiSeq** | **454** |
| Raw reads | 7827 (154) | 192 (4.2) |
| After removal of singletons | 4923 (99) | 126 (3.0) |
| After removal of non-classical alleles | 2447 (58) | 66 (1.6) |
| After error correction and removal of ‘poor’ amplicons | 2818 (62) | 63 (2.0) |
